# Patient consultation rate and clinical and NHS outcomes: a cross-sectional analysis of English primary care data from 2.7 million patients in 238 practices

**DOI:** 10.1186/s12913-019-4036-y

**Published:** 2019-04-06

**Authors:** Sarah Lay-Flurrie, Edouard Mathieu, Clare Bankhead, Brian D. Nicholson, Rafael Perera-Salazar, Tim Holt, F. D. Richard Hobbs, Chris Salisbury, on behalf of the NIHR School for Primary Care Research, Nuffield Department of Primary Care Health Sciences, University of Oxford

**Affiliations:** 1 Nuffield Department of Primary Care Health Sciences, University of Oxford, Radcliffe Primary Care Building, Radcliffe Observatory Quarter, Woodstock Road, Oxford, OX2 6GG UK; 2 Centre for Academic Primary Care, Bristol Medical School, University of Bristol, Bristol, UK

**Keywords:** General practice, Workload, Mortality, Quality of care, Hospitalization

## Abstract

**Background:**

Primary care workload is high and increasing in the United Kingdom. We sought to examine the association between rates of primary care consultation and outcomes in England.

**Methods:**

Cross sectional observational study of routine electronic health care records in 283 practices from the Clinical Practice Research Datalink from April 2013 to March 2014. Outcomes included mortality rate, hospital admission rate, Quality and Outcomes Framework (QOF) performance and patient satisfaction. Relationships between consultation rates (with a general practitioner (GP) or nurse) and outcomes were investigated using negative binomial and ordinal logistic regression models.

**Results:**

Rates of GP and nurse consultation (per patient person-year) were not associated with mortality or hospital admission rates: mortality incidence rate ratio (IRR) per unit change in GP/ nurse consultation rate = 1.01, 95% CI [0.98 to 1.04]/ 0.97, 95% CI [0.93 to 1.02]; hospital admission IRR per unit change in GP/ nurse consultation rate = 1.02, 95% CI [0.99 to 1.04]/ 0.98, 95% CI [0.94 to 1.032]. Higher rates of nurse but not GP consultation were associated with higher QOF achievement: OR = 1.91, 95% CI [1.39 to 2.62] per unit change in nurse consultation rate vs. OR = 1.04, 95% CI [0.87 to 1.24] per unit change in GP consultation rate. The association between the rates of GP/ nurse consultations and patient satisfaction was mixed.

**Conclusion:**

There are few associations between primary care consultation rates and outcomes. Previously identified demographic and staffing factors, rather than practice workload, appear to have the strongest relationships with mortality, admissions, performance and satisfaction. Studies with more detailed patient-level data would be required to explore these findings further.

**Electronic supplementary material:**

The online version of this article (10.1186/s12913-019-4036-y) contains supplementary material, which is available to authorized users.

## Background

Primary care workload in England increased by 16% between 2007 and 2014, [1] and similar large increases have been observed in secondary care [2]. The relationship between greater demand on primary care, or improved access, to primary care and outcomes such as secondary care use, mortality, satisfaction and quality of care is unclear.

Higher consultation rates might imply improved access to care with greater health care provision which could be associated with improvements in these outcomes. For example, patients in practices which provide more consultations might be less likely to use hospital services, owing to the fact that they do not experience difficulties accessing primary care. Alternatively, an increased consultation rate might indicate inadequate clinical triage and excessive practice workload. This might lead to a reduction in quality of care, worse patient outcomes and greater use of secondary health care resources. Such potential relationships are important for health service planning but are poorly understood.

Previous research has examined the association between continuity of care and hospital admissions, [3] but has largely been conducted at the patient level and has focussed on the route of admission [4, 5] or admission for specific conditions [6]. Similar research examining access to primary care and mortality has also been conducted at the patient level, [7] or in specific patients groups, [8] outside of the United Kingdom setting [9, 10]. Studies of quality of care have focussed on staffing levels, [11] which may fail to capture variation in time spent consulting rather than administrative tasks. Finally, studies of practice factors associated with patient satisfaction have not assessed consultation rates explicitly, but used data regarding patient experience of making an appointment, or list size and staff headcounts as proxies [12, 13].

We therefore explore the association between clinical and service outcomes (mortality, hospital admission rates, quality of care, and patient satisfaction) and consultation rates in primary care, at the general practice level.

## Methods

### Data sources

Consultation and patient demographic data were obtained from the Clinical Practice Research Datalink (CPRD), a research database of anonymised UK patient records [14]. English practices consenting to CPRD’s data linkage scheme were included in the study if they contributed data covering any part of the study period (1st April 2013 to 31st March 2014), and were defined as “up-to-standard” (CPRD definition of continuous high quality data recording fit for use in research).

CPRD data were linked to patient-level death registration data from the Office for National Statistics, inpatient Hospital Episodes Statistics data and Index of Multiple Deprivation (IMD) deprivation data. These datasets were further linked to practice-level data on staffing, [15] rurality, [16] patient satisfaction and quality of care. Patient satisfaction data were drawn from the General Practice Patient Survey (GPPS) [17]. Quality of care data were drawn from the Quality and Outcomes Framework (QOF), which is a financial incentive scheme that resources practices for performing certain evidence-based tasks in patients with chronic conditions [18, 19]. Patient level data was provided directly by CPRD and practice level data was downloaded from NHS digital (formerly the Health and Social Care Information Centre). The protocol for this study was approved by the Independent Scientific Advisory Committee (ISAC) of the Medicines and Healthcare Products Regulatory Agency (ISAC protocol number 15_120R).

### Data cleaning

Consultations in CPRD represent distinct openings of the clinical record, coded according to the type of contact, using 51 separate codes. These were grouped into the following consultation types: face-to-face surgery consultations, telephone contacts, home visits, administrative, and other. Equivalently, staff roles are coded using 67 separate codes, which we grouped into general practitioner (GP), nurse, other clinicians, and administrative roles [1]. This analysis was restricted to face-to-face, telephone or visit consultations with a GP or nurse. Patient level data for consultation, mortality and hospital admission rates were aggregated at the practice level (total number of GP/ nurse consultations, deaths and admissions). Data on age, gender and deprivation were also aggregated (percentage of patients who were female, percentage of patients aged over 64 and percentage of patients in the most deprived quintile of IMD). Continuous data on staffing, rurality, QOF performance and patient satisfaction data were grouped (e.g. into deciles) prior to linkage with patient-level data. This was a requirement of the Independent Scientific Advisory Committee (ISAC) to CPRD, to limit the possibility of identifying individual CPRD practices.

### Statistical analysis

Negative binomial regression models were used to examine the association between consultation rate and mortality/ hospital admission rates, with GP and nurse consultation rates examined separately. To account for patients and practices that only contributed data for part of the study period, the number of deaths/ admissions was modelled as the outcome variable with an offset term/ explanatory variable for the total patient person years of observation. Ordinal logistic regression models were used to examine the association between consultation rate and overall QOF performance score (decile, highest decile indicates best performance) and seven separate domains of patient satisfaction (decile, highest decile indicates greatest satisfaction, see Table 1).

**Table 1:** Domains of patient satisfaction from the GP Patient Survey

Patient satisfaction domain	
Able to get an appointment to see or speak to someone (% of patients responding “Yes”)	
Convenience of appointment (% of patients responding “Convenient”)	
How long until actually saw or spoke to GP / nurse (% of patients responding “Same or next day”)	
Is GP surgery currently open at times that are convenient (% of patients responding “Yes”)	
Ease of getting through to someone at GP surgery on the phone (% of patients responding “Easy”)	
Frequency of seeing preferred GP (% of patients responding “Always” or “Almost always”)	
Recommending GP surgery to someone (% of patients responding “Yes”)	

The following variables were included in all models, without selection: GP/nurse consultation rate (number of face-to-face, visit and telephone consultations with a GP/ nurse per patient person year), percentage of patients who were female, percentage of patients aged over 64, percentage of patients in the most deprived quintile of IMD, number of full-time equivalent (FTE) GPs/ nurses per 1000 patients, practice rurality (urban vs. rural) and practice training status (practice contains at least one trainee GP vs. none). List size was also included in the models for QOF performance and patient satisfaction, but not in the models for mortality/ admission rates due to collinearity with the person-years of observation offset term. Finally, the GPPS response rate was included in all models for patient satisfaction. For all models, complete case analysis was carried out. Factors were assessed for statistical significance at the 5% level.

## Results

In total, 316 English practices were eligible for inclusion in our study. There were missing data on the number of FTE GPs in two practices and on the number of FTE nurses in 31 practices, hence we analysed data from 283 practices (90%). Characteristics of these 283 practices are presented in Table 2 and according to tertile of consultation rate for GPs and nurses in Additional file 1 : Tables S1 and S2 respectively. The distribution of practices in each decile of QOF performance and GPPS domain are given in the online supplement (Additional file 1: Table S3).

**Table 2:** Practice characteristics (*N* = 283)

Variable	Mean (SD)/ N (%)
GP consultation rate (per patient person-year)	3.74 (1.24)
Nurse consultation rate (per patient person-year)	1.35 (0.78)
FTE GPs per 1000 patients	0.55 (0.16)
FTE Nurses per 1000 patients	0.25 (0.14)
Percentage of patients in most deprived quintile of IMD	16.7 (22.0)
Percentage of patients female	50.7 (1.50)
Percentage of patients over 64 years old	17.5 (6.16)
Number of deaths in 2013	68.2 (44.7)
Number of hospital admissions in 2013	2219 (1316)
List size	9808 (4717)
Patient person years of follow-up in 2013	8315 (4409)
Training practice (yes)	120 (42.4%)
Urban practice (yes)	237 (83.8%)

### Consultation rate, mortality and hospital admissions

Rate of consultation (per patient person-year) with a GP or nurse was not associated with mortality rate (incidence rate ratio (IRR) for GPs = 1.01, 95% CI [0.98 to 1.04]; IRR for nurses = 0.97, 95% CI [0.93 to 1.02]; Table 3). Factors associated with higher mortality rate were as follows: urban location (compared to rural), higher percentage of female patients, higher percentage of patients over 64, higher percentage of patients in the most deprived quintile, and a higher number of FTE GPs per 1000 patients (Table 3).

**Table 3:** Association between practice characteristics and mortality or hospital admission rate (adjusted results from negative binomial regression modelling)

	Mortality rate			Hospital admissions		
	IRR	95% CI		IRR	95% CI	
GP consultation rate (per patient person-year)	1.01	0.98	1.04	1.02	0.99	1.04
Nurse consultation rate (per patient person-year)	0.97	0.93	1.02	0.98	0.94	1.03
Percentage of patients aged over 64	1.06	1.05	1.07	1.02	1.02	1.03
Percentage of patients who are female	0.96	0.94	0.99	1.01	0.99	1.03
Number of FTE doctors per 1000 patients	1.55	1.22	1.97	1.05	0.86	1.29
Number of FTE nurses per 1000 patients	1.07	0.82	1.38	1.05	0.83	1.32
Urban location (compared to rural)	1.19	1.07	1.31	1.12	1.03	1.22
Percentage of patients in most deprived quintile of IMD	1.00	1.00	1.01	1.01	1.00	1.01
Training practice (yes compared to no)	1.04	0.97	1.11	0.95	0.90	1.01

Rate of consultation (per patient person-year) with a GP or nurse was not associated with hospital admission rate (incidence rate ratio (IRR) for GPs = 1.02, 95% CI [0.99 to 1.04]; IRR for nurses = 0.98, 95% CI [0.94 to 1.03]; Table 3). Factors associated with higher hospital admission rate were as follows: urban location (compared to rural), higher percentage of patients over 64 and higher percentage of patients in the most deprived quintile (Table 3).

### Consultation rate and quality of care assessed by QOF performance

The rate of nurse consultations was strongly associated with being in a higher achieving decile of QOF performance. An increase of one nurse consultation per patient year was associated with a 91% increase in odds of being in a higher achieving decile of QOF performance (OR = 1.91, 95% CI [1.39 to 2.62]). The only other factor associated with higher QOF performance was having a higher percentage of patients aged 64 years or over. Rate of GP consultation (per patient person-year) was not associated with being in a higher achieving decile QOF performance (OR = 1.04, 95% CI [0.87 to 1.24], Table 4).

**Table 4:** Association between practice characteristics and decile of QOF achievement (adjusted results from ordinal logistic regression modelling)

	OR	95% CI	
GP consultation rate (per patient person-year)	1.04	0.87	1.24
Nurse consultation rate (per patient person-year)	1.91	1.39	2.62
List size (per 1000 patients)	1.05	1.00	1.10
Percentage of patients aged over 64	1.05	1.01	1.10
Percentage of patients who are female	0.94	0.82	1.09
Number of FTE doctors per 1000 patients	1.10	0.25	4.90
Number of FTE nurses per 1000 patients	0.21	0.02	1.80
Urban location (compared to rural)	1.06	0.57	1.98
Percentage of patients in most deprived quintile of IMD	1.00	0.99	1.01
Training practice (yes compared to no)	1.32	0.83	2.09

### Consultation rate and patient satisfaction

Rate of nurse consultation (per patient person-year) was not associated with any of the patient satisfaction domains studied (Additional file 1, Tables S2 and S3). Rate of GP consultation was associated with being in a higher achieving decile of the following patient satisfaction domains: the proportion of people who would recommend their GP surgery to someone (OR = 1.45, 95% CI [1.19 to 1.77]); the proportion of patients able to see or speak to someone on the same or next day (OR = 1.31, 95% CI [1.06 to 1.60]); and the proportion of people who think the surgery is open at convenient times (OR = 1.44, 95% CI [1.17 to 1.78]). In five of the seven domains of satisfaction studied, practices with larger list sizes were more likely to have lower patient satisfaction (Additional file 1: Tables S4 and S5). Other practice characteristics had less consistent associations with each of the measures of satisfaction (Additional file 1: Tables S4 and S5).

## Discussion

We have found that rates of GP and nurse consultation are not associated with mortality or hospital admission rates at the practice level. Higher rates of nurse consultation are associated with higher QOF achievement, and higher rates of GP consultation are associated with measures of patient satisfaction with respect to access. Other factors which have previously been observed to relate to these outcomes, such as age, deprivation and urban location, were more influential than practice workload.

### Strengths and limitations

A strength of our analysis is the use of data from multiple different sources to describe practice characteristics and outcomes. Practices contributing data to the CPRD are known to be representative of the UK population, so our results can be considered generalizable.

A limitation of our analysis is the grouping of certain outcome variables (such as QOF performance) into deciles, which was a requirement of the ethical approval for this study. This may have limited our ability to distinguish between practices and detect weaker associations. We have also studied several outcomes, particularly with respect to patient satisfaction, so these findings should be interpreted with caution, although they have face validity. Further studies are required to confirm our findings.

In our analyses, we adjusted for the number of GPs/ nurses per 1000 patients and characteristics of the patient population. Hence a difference in consultation rates in our models can be considered to reflect a difference in the extent to which practices, with equivalent staffing capacity and equivalent patient demand, are meeting patient demand. Our finding that there is no relationship between consultation rates and mortality or hospitalisation may therefore be surprising. This may be due to a cancelling out of both positive and negative effects but it may also indicate that any unmet patient demand relates to acute, self-limiting conditions. Disentangling these effects would require different research designs and detailed information about appointment availability and presenting problem.

Conversely, it is also possible that consultation rate is an indicator of the number of appointment slots available (supply) rather than ability to meet demand. To explore this we did repeat our analyses, removing variables for the number of GPs/ nurses per 1000 patients from the models and hence allowing consultation rate to act as a proxy for supply. Results for all outcomes were similar, with no association between consultation rates and outcomes observed in most cases (data not shown). This indicates that regardless of whether general practice workload is viewed as a problem of demand or supply, it’s association with outcomes is mixed and limited when compared with demographic factors. It may also suggest that after accounting for these demographic factors, the availability of consultations is such that the chance of death or hospitalisation is consistent across England and the service provided is broadly equitable.

We summarized data and conducted our analysis at the practice level, rather than using multilevel modelling approaches using both patient and practice level data. This was in part due to computational difficulties of conducting multilevel modelling on such a larger number of observations (data from more than 2.7 million patients has been summarized at the practice level). Furthermore, we were primarily interested in examining the relationships between consultation, mortality and admission rates to inform practice management and health policy. Arguably, examining these relationships at the patient level would answer a different research question.

### Comparisons with the literature

Our finding that practices with a higher number of FTE GPs per 1000 patients also have higher mortality rates may be surprising. One possible explanation is that practices employ more doctors in settings where the patient population has greater health needs. Although we adjusted for practice deprivation and patient age we did not have more sensitive measures of patient complexity or morbidity. Alternatively, a greater number of available GPs may have an adverse effect of reducing continuity of care which has been linked to mortality [7] and hospital admissions, [3] which may also explain our findings. Finally, it is possible that this is a chance finding because of the number of statistical tests undertaken, and it needs replication in further studies using different datasets.

We did not find an association between consultation rates and hospital admission rates. A 2013 study [5] found that practices providing more timely access to primary care had fewer self-referred emergency department visits. Although we were not able to measure when appointments took place in relation to time of initial contact and did not investigate route of admission, our results suggest that any effect of reduced primary care access on accident and emergency attendances does not propagate through to admissions.

Our findings build on a previous study examining quality of care and nurse staffing levels which found that a higher ratio of nurses per patient was associated with better performance in clinical indicators from the QOF [11]. In contrast, we found no associations between practice nurse staffing and any of the outcomes examined, with the exception of the relationship between nurse consultation rate (adjusted for the number of nurses per patient) and QOF performance. This may suggest that this relationship relies on nurses having good availability to conduct consultations, rather than performing administrative tasks.

Our finding that higher patient list size is associated with poorer patient satisfaction is consistent with a study from 1995 [13]. This suggests that despite numerous changes to the primary care system in the last 10 years, larger practices may inherently struggle to satisfy their patients.

### Implications for research and practice

We have previously shown that general practice consultation rates have increased substantially in recent years, such that practices appear to be reaching saturation point [1]. This investigation, which was largely exploratory and hypothesis generating, explored whether there is an association between practice consultation rates and patient outcomes. The fact that few associations were observed between consultation rates and outcomes could be considered reassuring.

There have been many policy initiatives in recent years to improve access to GP consultations, for example through the extended hours access scheme [20] and through greater use of telephone consultations, [21] and these initiatives are likely to increase consultation rates. Our study suggests that providing more primary care consultations is not associated with improvements in any of the important patient outcomes that we studied. The implication is that policy should focus more on factors which are clearly associated with improved outcomes, such as supporting practices in deprived or urban areas or with elderly populations, more than focusing on appointment availability and consultation rate per se. Furthermore, practice size is inversely related to patient satisfaction, raising questions about the current policy to encourage larger practices or setting a challenge to bigger practices in how to maintain patient continuity with GPs.

This study demonstrates that greater provision of nurse consultations is associated with improvements in practice QOF performance. For long term conditions, where evidence is strong enough to determine clear treatment pathways as with QOF, our findings provide support for the policy of encouraging more nurses and allied health professionals into general practice, [22] particularly if they dedicate their time to direct patient care.

It might be argued that this study suggests that practices can work harder and offer more consultations while still maintaining good patient outcomes. This is reassuring for patients at a time of rapidly rising demand in primary care. However, this scenario may have negative consequences for health professionals themselves, with many GPs leaving practice early in part because they find it impossible to provide good care in the face of ever increasing demand [23]. With 40% of GPs in South West England reporting their intention to retire within 5 years, mainly citing workload and working conditions, [24] there can be no complacency over these reassuring data that workload increases have not yet been associated with worsening patient outcomes. The impact of initiatives announced by the NHS to boost GP numbers and improved terms await determination.

## Conclusions

Using linked, routinely collected data from primary and secondary care in England, we have shown that rates of consultation with a GP or nurse are not associated with clinical outcomes at the general practice level. However, higher rates of consultation are associated with greater quality of care and measures of patient satisfaction. Our results can be viewed as reassuring, indicating that despite increasing pressure in general practice, this is not associated with negative patient outcomes. However, they also indicate that improved clinical outcomes may not be achieved by simply increasing the number of consultations offered in general practice. A more nuanced approach, taking into account the particular demographic challenges individual practices face and concerns of professionals regarding workload, may be warranted.

## Additional file

Additional file 1: Supplementary results. This file contains additional tables presenting results from the analyses which could not be included in the main manuscript. (DOCX 29 kb)

## Abbreviations

CPRD: Clinical Practice Research Datalink
FTE: Full-time equivalent
GP: General practitioner
GPPS: General Practice Patient Survey
IMD: Index of multiple deprivation
ISAC: Independent Scientific Advisory Committee
QOF: Quality and Outcomes Framework
