# Supplementary material for: Patient consultation rate and clinical and NHS outcomes: a cross-sectional analysis of English primary care data from 2.7 million patients in 238 practices
Source: BMC Health Serv Res. 2019 Apr 6;19:219. doi: 10.1186/s12913-019-4036-y (PMC6451312; doi:10.1186/s12913-019-4036-y)
Supplement: Supplementary file 1 — Supplementary results. This file contains additional tables presenting results from the analyses which could not be included in the main manuscript. (DOCX 29 kb) [file 12913_2019_4036_MOESM1_ESM.docx]

Additional File 1

Table S1: Practice characteristics by tertile of GP consultation rate

|  | Mean (SD)/ N (%) | | |
| --- | --- | --- | --- |
|  | Tertile of GP consultation rate (per person-year) | | |
| Variable | < 3.25 (N=91) | 3.25 to 4 (N=97) | 4 to 12.9 (N=95) |
| GP consultation rate (per patient person-year) | 2.60 (0.61) | 3.61 (0.23) | 4.98 (1.18) |
| Nurse consultation rate (per patient person-year) | 1.15 (0.71) | 1.41 (0.80) | 1.47 (0.80) |
| FTE GPs per 1000 patients | 0.51 (0.16) | 0.56 (0.13) | 0.59 (0.16) |
| FTE Nurses per 1000 patients | 0.24 (0.10) | 0.25 (0.11) | 0.27 (0.19) |
| Percentage of patients in most deprived quintile of IMD | 16.3 (20.8) | 17.9 (22.8) | 16.0 (22.4) |
| Percentage of patients female | 50.7 (1.82) | 50.6 (1.49) | 50.7 (1.13) |
| Percentage of patients over 64 years old | 16.3 (6.19) | 17.3 (6.04) | 18.7 (6.09) |
| Number of deaths in 2013 | 54.0 (36.3) | 75.1 (49.7) | 74.6 (43.8) |
| Number of hospital admissions in 2013 | 1868 (1117) | 2521 (1505) | 2247 (1215) |
| List size | 8936 (4106) | 10546 (5166) | 9889 (4697) |
| Patient person years of follow-up in 2013 | 7312 (4068) | 9320 (4962) | 8249 (3913) |
| Training practice (yes) | 28 (30.8%) | 51 (52.6%) | 41 (43.2%) |
| Urban practice (yes) | 82 (90.1%) | 84 (86.6%) | 71 (74.7%) |

Table S2: Practice characteristics by tertile of nurse consultation rate

|  | Mean (SD)/ N (%) | | |
| --- | --- | --- | --- |
|  | Tertile of nurse consultation rate (per person-year) | | |
| Variable | < 0.90 (N=93) | 0.90 to 1.56 (N=91) | 1.56 to 3.80 (N=99) |
| GP consultation rate (per patient person-year) | 3.31 (1.06) | 3.86 (1.10) | 4.05 (1.41) |
| Nurse consultation rate (per patient person-year) | 0.56 (0.28) | 1.20 (0.19) | 2.21 (0.52) |
| FTE GPs per 1000 patients | 0.54 (0.16) | 0.52 (0.12) | 0.59 (0.17) |
| FTE Nurses per 1000 patients | 0.20 (0.09) | 0.23 (0.08) | 0.32 (0.19) |
| Percentage of patients in most deprived quintile of IMD | 21.7 (23.5) | 12.3 (19.7) | 16.1 (21.7) |
| Percentage of patients female | 50.5 (1.84) | 50.8 (1.22) | 50.7 (1.36) |
| Percentage of patients over 64 years old | 15.1 (5.82) | 17.6 (5.66) | 19.6 (6.19) |
| Number of deaths in 2013 | 54.0 (39.7) | 74.5 (41.8) | 75.5 (48.6) |
| Number of hospital admissions in 2013 | 1898 (1183) | 2404 (1255) | 2350 (1442) |
| List size | 8872 (4194) | 10299 (4459) | 10236 (5294) |
| Patient person years of follow-up in 2013 | 7311 (4074) | 9200 (4408) | 8444 (4563) |
| Training practice (yes) | 30 (32.3%) | 46 (50.6%) | 44 (44.4%) |
| Urban practice (yes) | 86 (92.5%) | 75 (82.4%) | 76 (76.8%) |

Table S3: Percentage of practices in each decile of QOF performance or GPPS domain (N=283)

|  |  | GPPS satisfaction measures | | | | | | |  |  |
| --- | --- | --- | --- | --- | --- | --- | --- | --- | --- | --- |
| Decile | QOF achievement | Able to get an appointment to see or speak to someone (% "Yes"), decile | Convenience of appointment (% "Convenient"), decile | How long until actually saw or spoke to GP / nurse (% "Same or next day"), decile | Is GP surgery currently open at times that are convenient (% "Yes"), decile | Ease of getting through to someone at GP surgery on the phone (% "Easy"), decile | Frequency of seeing preferred GP (% "Always" or "Almost always"), decile | Recommending GP surgery to someone who has just moved to area (% "Yes"), decile | GPPS Response rate (%), decile | Long-standing health condition (% "Yes"), decile |
| 1 | 4.2 | 6.7 | 9.5 | 10.3 | 12.4 | 10.6 | 12.4 | 8.8 | 2.5 | 8.1 |
| 2 | 7.1 | 11.0 | 10.3 | 11.7 | 11.0 | 12.0 | 9.2 | 7.8 | 7.1 | 13.1 |
| 3 | 5.7 | 9.9 | 12.0 | 9.2 | 9.5 | 11.3 | 11.7 | 11.3 | 7.4 | 6.7 |
| 4 | 8.1 | 12.0 | 13.4 | 13.1 | 15.2 | 10.3 | 10.6 | 8.8 | 8.8 | 15.2 |
| 5 | 11.3 | 8.8 | 8.5 | 12.0 | 8.8 | 12.0 | 9.5 | 10.3 | 8.1 | 10.6 |
| 6 | 9.2 | 10.3 | 8.5 | 7.8 | 10.3 | 11.3 | 12.0 | 7.4 | 14.8 | 9.2 |
| 7 | 8.8 | 11.0 | 15.9 | 8.8 | 7.8 | 8.1 | 10.6 | 15.2 | 13.8 | 10.3 |
| 8 | 14.8 | 12.4 | 7.4 | 9.2 | 11.3 | 8.8 | 6.0 | 12.0 | 17.3 | 11.3 |
| 9 | 12.4 | 9.9 | 6.7 | 9.9 | 5.3 | 8.5 | 8.1 | 9.9 | 9.5 | 5.3 |
| 10 | 18.0 | 8.1 | 7.8 | 8.1 | 8.5 | 7.1 | 8.8 | 8.5 | 10.6 | 10.3 |
| Missing | 0.4 | 0.0 | 0.0 | 0.0 | 0.0 | 0.0 | 1.1 | 0.0 | 0.0 | 0.0 |

Table S4: Association between practice characteristics and decile of patient satisfaction measures related to access (adjusted results from ordinal logistic regression modelling).

|  | Ease of getting through to someone at GP surgery on the phone (% "Easy") | | | Frequency of seeing preferred GP (% "Always" or "Almost always") | | | Able to get an appointment to see or speak to someone (% "Yes") | | | How long until actually saw or spoke to GP / nurse (% "Same or next day") | | |
| --- | --- | --- | --- | --- | --- | --- | --- | --- | --- | --- | --- | --- |
|  | OR | 95% CI | | OR | 95% CI | | OR | 95% CI | | OR | 95% CI | |
| List size (per 1000 patients) | 0.86 | 0.81 | 0.91 | 0.93 | 0.88 | 0.98 | 0.95 | 0.90 | 1.00 | 0.97 | 0.92 | 1.02 |
| GP consultation rate (per patient person-year) | 1.20 | 0.99 | 1.45 | 1.15 | 0.96 | 1.38 | 1.16 | 0.96 | 1.41 | 1.31 | 1.06 | 1.60 |
| Nurse consultation rate (per patient person-year) | 1.11 | 0.82 | 1.50 | 0.81 | 0.59 | 1.12 | 1.11 | 0.83 | 1.50 | 1.01 | 0.74 | 1.39 |
| Percentage of patients aged over 64 | 0.97 | 0.92 | 1.03 | 1.00 | 0.95 | 1.06 | 1.02 | 0.96 | 1.08 | 0.98 | 0.93 | 1.04 |
| Percentage of patients who are female | 0.83 | 0.71 | 0.96 | 0.90 | 0.77 | 1.06 | 0.98 | 0.84 | 1.15 | 0.95 | 0.82 | 1.10 |
| Number of FTE doctors per 1000 patients | 1.65 | 0.39 | 7.04 | 1.20 | 0.26 | 5.60 | 2.89 | 0.65 | 12.74 | 0.76 | 0.17 | 3.37 |
| Number of FTE nurses per 1000 patients | 0.55 | 0.11 | 2.86 | 1.96 | 0.38 | 10.18 | 0.77 | 0.15 | 3.94 | 5.10 | 0.81 | 32.06 |
| Urban location (compared to rural) | 0.62 | 0.30 | 1.27 | 0.77 | 0.38 | 1.55 | 0.89 | 0.44 | 1.80 | 0.72 | 0.37 | 1.42 |
| Percentage of patients in most deprived quintile of IMD | 0.99 | 0.97 | 1.00 | 0.99 | 0.98 | 1.01 | 0.97 | 0.96 | 0.98 | 1.02 | 1.01 | 1.04 |
| Training practice (yes compared to no) | 0.72 | 0.45 | 1.17 | 0.41 | 0.25 | 0.66 | 0.80 | 0.50 | 1.27 | 1.41 | 0.89 | 2.24 |
|  |  |  |  |  |  |  |  |  |  |  |  |  |
| GPPS response rate decile (1 = lowest response rate) |  |  |  |  |  |  |  |  |  |  |  |  |
| 2 | 0.31 | 0.06 | 1.60 | 0.42 | 0.07 | 2.36 | 0.43 | 0.07 | 2.63 | 2.33 | 0.46 | 11.86 |
| 3 | 0.44 | 0.08 | 2.41 | 1.04 | 0.18 | 5.87 | 0.38 | 0.06 | 2.42 | 4.26 | 0.84 | 21.68 |
| 4 | 0.50 | 0.09 | 2.78 | 1.78 | 0.30 | 10.45 | 0.59 | 0.09 | 3.81 | 3.96 | 0.72 | 21.76 |
| 5 | 0.35 | 0.06 | 2.11 | 0.80 | 0.12 | 5.23 | 0.25 | 0.04 | 1.79 | 5.89 | 0.98 | 35.27 |
| 6 | 0.45 | 0.08 | 2.57 | 2.09 | 0.34 | 12.89 | 0.53 | 0.08 | 3.62 | 5.64 | 1.03 | 30.95 |
| 7 | 0.74 | 0.12 | 4.64 | 3.11 | 0.48 | 20.00 | 0.71 | 0.10 | 5.23 | 6.26 | 1.06 | 36.94 |
| 8 | 0.63 | 0.10 | 4.04 | 2.72 | 0.40 | 18.49 | 0.54 | 0.07 | 4.02 | 7.20 | 1.17 | 44.17 |
| 9 | 0.74 | 0.10 | 5.33 | 3.10 | 0.40 | 23.95 | 0.66 | 0.08 | 5.56 | 8.34 | 1.23 | 56.54 |
| 10 | 1.46 | 0.17 | 12.39 | 6.66 | 0.72 | 61.63 | 1.00 | 0.10 | 9.62 | 14.28 | 1.84 | 110.83 |

Table S5: Association between practice characteristics and decile of patient satisfaction measures related to convenience and recommendations (adjusted results from ordinal logistic regression modelling).

|  | Convenience of appointment (% "Convenient") | | | Is GP surgery currently open at times that are convenient (% "Yes") | | | Recommending GP surgery to someone who has just moved to area (% "Yes") | | |
| --- | --- | --- | --- | --- | --- | --- | --- | --- | --- |
|  | OR | 95% CI | | OR | 95% CI | | OR | 95% CI | |
| List size (per 1000 patients) | 0.93 | 0.88 | 0.98 | 0.94 | 0.89 | 0.99 | 0.92 | 0.87 | 0.97 |
| GP consultation rate (per patient person-year) | 1.12 | 0.92 | 1.36 | 1.44 | 1.17 | 1.78 | 1.45 | 1.19 | 1.77 |
| Nurse consultation rate (per patient person-year) | 0.94 | 0.69 | 1.28 | 1.13 | 0.82 | 1.54 | 0.93 | 0.67 | 1.27 |
| Percentage of patients aged over 64 | 1.05 | 0.99 | 1.11 | 1.09 | 1.03 | 1.16 | 0.99 | 0.94 | 1.05 |
| Percentage of patients who are female | 0.84 | 0.72 | 0.97 | 0.85 | 0.72 | 1.00 | 0.95 | 0.81 | 1.11 |
| Number of FTE doctors per 1000 patients | 0.96 | 0.22 | 4.17 | 0.72 | 0.16 | 3.33 | 0.86 | 0.20 | 3.74 |
| Number of FTE nurses per 1000 patients | 2.42 | 0.41 | 14.11 | 5.96 | 0.69 | 51.26 | 1.36 | 0.23 | 8.07 |
| Urban location (compared to rural) | 0.48 | 0.23 | 1.00 | 0.77 | 0.36 | 1.62 | 0.56 | 0.27 | 1.15 |
| Percentage of patients in most deprived quintile of IMD | 1.00 | 0.99 | 1.01 | 1.00 | 0.99 | 1.02 | 0.99 | 0.98 | 1.00 |
| Training practice (yes compared to no) | 1.37 | 0.85 | 2.21 | 1.33 | 0.82 | 2.15 | 1.09 | 0.67 | 1.76 |
|  |  |  |  |  |  |  |  |  |  |
| GPPS response rate decile (1 = lowest response rate) |  |  |  |  |  |  |  |  |  |
| 2 | 0.63 | 0.12 | 3.27 | 0.15 | 0.03 | 0.75 | 0.24 | 0.05 | 1.24 |
| 3 | 0.70 | 0.13 | 3.74 | 0.13 | 0.03 | 0.73 | 0.24 | 0.04 | 1.28 |
| 4 | 1.54 | 0.28 | 8.42 | 0.15 | 0.03 | 0.77 | 0.57 | 0.10 | 3.15 |
| 5 | 1.08 | 0.18 | 6.38 | 0.07 | 0.01 | 0.41 | 0.14 | 0.02 | 0.82 |
| 6 | 1.14 | 0.20 | 6.37 | 0.12 | 0.02 | 0.68 | 0.38 | 0.07 | 2.15 |
| 7 | 1.74 | 0.29 | 10.59 | 0.14 | 0.02 | 0.83 | 0.76 | 0.13 | 4.51 |
| 8 | 1.24 | 0.20 | 7.75 | 0.15 | 0.02 | 0.90 | 0.85 | 0.14 | 5.26 |
| 9 | 1.48 | 0.21 | 10.38 | 0.17 | 0.03 | 1.18 | 0.55 | 0.08 | 3.90 |
| 10 | 1.15 | 0.14 | 9.31 | 0.09 | 0.01 | 0.76 | 1.34 | 0.16 | 11.34 |
